# Converging economies of care? Immigrant women workers across 17 countries and four care regimes

**DOI:** 10.1177/00221856231221639

**Published:** 2023-12-19

**Authors:** Naomi Lightman

**Affiliations:** Associate Professor of Sociology, 7984 Toronto Metropolitan University, 350 Victoria St, Toronto, ON, Canada

**Keywords:** Care work, care regime, cross-national comparison, immigration, gender

## Abstract

This study analyses 17 care economies using 2016 Luxembourg Income Study data to contribute to extant debate regarding the ongoing utility of care regimes as a classificatory schema for cross-national comparison. Examining similarities and differences in the provision of low-status work in health, education, social work, and domestic services – the ‘care economy’ – the data reveal devaluation of the labour done by immigrant women care workers, net of national and regime-level variation. In addition, numerous similarities across liberal, corporatist, social democratic, and central and eastern European care regimes emerge, in terms of the overrepresentation of immigrant women in low status care work, and the disproportionate financial penalties these workers incur. Together, findings suggest that notwithstanding national and policy-specific differences, there has been considerable convergence across economies of care towards a ‘migrant in the market’ model of employment. Such large-scale evidence of this trend calls into question the ongoing efficacy of care regimes for national comparisons of migrant care work under conditions of neoliberal globalization.

## Introduction

Cross-national studies of social care provisioning, or ‘care work’, inevitably grapple with the predominance of Esping-Anderson's seminal typology of welfare regimes in *The Three Worlds of Welfare Capitalism* (1990), with critics cautioning of the need ‘to be sceptical of the regime concept altogether as a tool for comparative analysis’ (Kasza, 2002, p. 292). Yet *care regimes*, a feminist adaptation of Esping-Anderson's classificatory schema, remain a popular alternative used to facilitate cross-national comparisons of care economies (e.g. Albertini, 2014; Lightman, 2021). Care regimes are thought to better recognize the gendered nature of this labour, the unique relationship between the family and state in providing social care, and the increasing reliance on immigrant women as paid care providers (Ranci, Cordini, Cerea & Arlotti, 2021; Schulz-Nieswandt, Jochimsen, Maier-Rigaud & Kurscheid, 2006).

However, in recent decades, scepticism has also emerged regarding the ongoing utility of the concept of care regimes under conditions of neoliberal globalization (Baines, Charlesworth & Daly, 2016; Pailhé, Solaz & Stanfors, 2021). Specifically, it is suggested that there has been a global convergence in the provisioning of health and domestic services across Global North and Global South countries, in terms of the commodification of care services and the increasing reliance on a ‘migrant in the market’ model of care provisioning (Pailhé et al., 2021; Van Hooren, 2012). Such scholars also note that few national care economies exhibit the internal consistency necessary to validate the care regime concept, and instead propose that policy-specific comparisons may be a more promising avenue for comparative research (Cangiano, 2009; Kasza, 2002).

The present study takes up this debate with a specific focus on immigrant women care workers, examining 17 countries and four care regimes, and using 2016 Luxembourg Income Study (LIS) national micro-data. The aim is to provide a large-scale detailed empirical investigation of the state of the paid care economy at the national and the regime levels, with specific attention paid to the outcomes of immigrant women care workers. As well, rather than viewing gender and immigration status as independent categories of analysis, as has been done in most previous quantitative cross-national comparisons (e.g. King-Dejardin, 2019; Lightman, 2021), here I build on qualitative studies that apply an intersectional lens, and foreground any unique outcomes for immigrant women care workers in particular, in a context of gendered, racialized and classed systems of domination in low-value work (Banerjee, 2019; Tungohan, 2019). In doing so, this paper takes up the following three central research questions:

1. Do care economies exhibit major differences between and within care regimes? Or, have the forces of globalization fostered substantial convergence towards a ‘migrant in the market’ model of employment?
2. Are women immigrants overrepresented in the care workforce cross-nationally? And is this true regardless of educational attainment?
3. Are similar wage penalties experienced by care workers across countries and care regimes? And further, do these care wage penalties particularly disadvantage women immigrants, when controlling for country-level variation and care regime type?

The paper begins with a review of the literature on care regimes, including details regarding standard characteristics of the four regimes examined. This is followed by an assessment of existing critiques that question the ongoing utility of care regimes as a heuristic measure to assess similarities and differences in care provisioning at the cross-national level. Next, I present the empirical findings, including discussion of how I operationalize low status care work, a detailed examination of the state of the care economies, and data on the representation of immigrant women care workers along with their comparative wages. Using descriptive and multivariate analyses, including logistic and ordinary least squares (OLS) regressions with interactive effects, I maintain a singular focus on the employment of and outcomes for immigrant women workers. Findings: (a) support those who suggest that care regimes are converging towards a ‘migrant in the market’ model of care provision, as well as (b) bolstering qualitative intersectional analyses that demonstrate the particular vulnerability and devaluation of labour done by immigrant women in low status caring occupations. The paper concludes with a discussion of data limitations and the suggestion that we are trending towards a care convergence with respect to immigrant care workers across care regimes, with associated concerns regarding the treatment of these essential workers and the broader accessibility of social care provisioning.

## Care regimes and the devaluation of low wage care

Care work is typically defined as interactive labour assisting children, the elderly, or people with complex healthcare needs (England, Budig & Folbre, 2002). It has several key characteristics internationally. First, is the feminization of this (paid and unpaid) workforce, along with the association of care work with ‘women's work’ and its social and financial devaluation (Duffy & Armenia, 2021; Folbre, 2012; McDonald, Thorpe & Irvine, 2018). Second, is the increasing reliance on immigrant women workers to do the ‘dirty’, difficult, and dangerous (3D) jobs within the paid care economy^1^, with many negative implications to workers’ physical and mental health (England et al., 2002; Hochschild, 2000). And third, is the high rates of precarious and part-time labour in many caring occupations that are deemed ‘low status’ (e.g. personal support work and care provided in private homes), along with attendant low wages (Budig & Misra, 2010; Hopfgartner, Seubert, Sprenger & Glaser, 2022).

Despite general consensus with respect to the above key characteristics of care work, scholars are divided with regard to the ongoing utility of *care regimes* as a heuristic mechanism to conceptualize and measure similarities and differences in the provisioning of care cross-nationally (Fagertun, 2017; Lightman, 2021). Defined as the ‘caring dimension of the welfare state’ (Knijn & Kremer, 1997, p. 328), where care providers and recipients are the focus of analysis, care regimes emerged in the 1990s as a feminist evolution of Esping-Anderson's (1990) seminal typology of welfare regimes (Giordano, 2022). Subsequently, care regimes have been classified based on a variety of criteria. Yet often, the concept of ‘familialism’ is used to distinguish between care regimes with more and less interventive policies in health and social services (Albertini, 2014; Kadi, Rodrigues, Kahlert, Hofmann & Bauer, 2022; Lightman, 2019). Other care regime scholars focus on childcare vs. elder care, or on the breakdown of formal (paid) vs. informal (unpaid) care within the economy (Giordano, 2022). Yet across any differences in operationalization, supporters of care regime typologies suggest that they allow for both the specificities of national care economies and for the convenient ‘bundling’ of countries together which display similar characteristics, in terms of the degree of commodification and stratification, and/or the relative reliance on immigrant women workers in the care economy (Van Hooren, 2012).

The present study examines 17 countries that are typically grouped into four ‘ideal-typical’ care regimes, building off of the classificatory schemas provided by Van Hooren (2012), King-Dejardin (2019) and Lightman (2021). These scholars focus on the principal institution providing care: namely, the state, market, family, or a mix. The defining characteristics of each of these care regimes are briefly reviewed below, with particular attention paid to the employment of immigrant women within lower status care occupations.

1. *Liberal* care regimes (e.g. the United States, the United Kingdom) are those most often associated with a free market ideology. Here, care services are primarily purchased in the private market by individuals, with no universal access guaranteed at the state level (Folbre, 2012; Martinez-Lacoba, Pardo-Garcia & Escribano-Sotos, 2021). Large components of the care economy are outsourced to private agencies, with immigrant (women) workers a ‘structural feature’ of the care economy (Cangiano, 2009, p. 163). Liberal care regimes have thus been characterized in prior research as having a ‘migrant in the market’ (Van Hooren, 2012, p. 138) model of employment, due to the heavy reliance on immigrant women workers to engage in low-status, low-wage forms of (typically privatized) care. Immigrant workers also often incur additional wage penalties compared to non-immigrant care workers (Lightman, 2019; Turnpenny & Hussein, 2022).
2. *Corporatist* care regimes (e.g. Germany, France) have a stronger reliance on ‘familialism’, or the traditional male breadwinner model of welfare provisioning, than liberal care regimes, with a gendered emphasis on women in private homes providing care (Shire, 2015). However, such countries continue to have more developed welfare states than in many other care regimes, often including state-run policies such as supportive parental leave, paid sick leave, child benefits, and childcare services (Isakjee, 2017; King-Dejardin, 2019). Corporatist care regimes thus enjoy some worker protections due to a stronger role for unions and state oversight than in liberal care regimes. However, previous research has found that they similarly have a heavy reliance on immigrants in low-wage and private-sector care jobs, similar to the ‘migrant in the market’ model (Lightman, 2021).
3. Next, and in direct contrast to liberal regimes, *social democratic* care regimes (e.g. Denmark, the Netherlands) provide universal access to care services, independent of income or family circumstances. Such countries are thought to emphasize the de-familialization of care work, prioritizing gender equality and the facilitation of the dual-earner model (Van Hooren, 2012). Social democratic regimes provide care largely or entirely within the public sector, where such jobs are well paid, unionized, and secure/permanent. Thus, in this regime, care work jobs are better paid and more attractive to native-born workers than elsewhere, decreasing demand for immigrant labour within the care economy (Eydal & Rostgaard, 2016; Giordano, 2022; Rostgaard, 2014).
4. *Central and eastern European (CEE)* care regimes (e.g. the Czech Republic, Poland) have a strong emphasis on privatization of risk and family responsibility for care, similar to many southern European nations (Schulz-Nieswandt et al., 2006). Many countries included in the CEE regime are transitioning from former socialist governance and are enduring severe fiscal restrictions resulting in a care deficit, alongside social security systems that traditionally had a strong state role in provisioning. The result is growing privatization, with institutional services often insufficient, expensive, and accessible only for the middle-class (Hacker, 2009). Notably, some of these countries are a source for immigrant care workers, while the relatively wealthier states within this regime import immigrant domestic workers to address limitations in state provisioning. Thus, existing research suggests considerable country-level variation within the CEE regime type, but also an overall shift towards a more liberal-style of care provisioning (Bartha & Zentai, 2020).

Notably, promoters of care regimes emphasize that these are only ‘ideal types’ used for comparative welfare analyses, and that individual countries inevitably differ in the precise structuring of their systems of social security and do not need to have identical key characteristics to be included and grouped together (Aspalter, 2012). Such scholars also note that care regimes classifications are not ‘water-tight’ and that there is variability across schemas, as countries are often classified differently across different studies (see Giordano, 2022, for an excellent overview). Thus, supporters of care regimes recognize national variation, while highlighting the utility of this heuristic measure to organize cross-national comparisons of care (Giordano, 2022; Van Hooren, 2012).

However, as the following section details, care regimes are not without critics. Evidence of increasing austerity measures worldwide and the globalization of care markets bolster suggestions that the ‘migrant in the market’ model, typical to liberal care regimes, is becoming predominant internationally (Baines et al., 2016; Parreñas, 2020). This line of reasoning suggests that care regimes may ultimately be less useful for analyses of immigrant women care workers.

## A transnational economy of care: market-driven convergence towards a ‘migrant in the market’ model

In contrast to scholars who articulate the ongoing utility of care regimes as a classificatory mechanism (Bartha & Zentai, 2020; Kadi et al., 2022), there are those who instead focus on a growing transnational economy of care, detailing how and why care work (and care workers) are moving towards a trend of international convergence (Baines et al., 2016; Baines, Dulhunty & Charlesworth, 2021; Pailhé et al., 2021). Here, the suggestion is made that due to widespread shifts towards increasing austerity measures and the retrenchment of welfare state provisioning across both Global North and Global South countries, care regimes are becoming less relevant and/or even obsolete. Instead, the argument is made that there is a general trend towards a more ‘liberal’ style of care provisioning, with governments worldwide prioritizing free market ideologies and privatizing care services (Abrahamson, 2017; Brimblecombe, Pickard, King & Knapp, 2018; Lightman, 2021).

In conjunction with such international ‘policies of constraint’ (Baines et al., 2016, p. 449), has been the increasing prevalence and recognition of the ‘global care chain’ (Hochschild, 2000). This care chain facilitates immigrant women workers providing low wage care across borders, typically in the private, informal, and for-profit sectors, and often without the option of permanent resettlement in their countries of employment (Hayes & Hartmann, 2017; Turnpenny & Hussein, 2022). The global care chain relies on the continued high demand for paid care work due to aging populations in many wealthy nations, with female immigrant workers providing a convenient market-based solution to national labour market shortages (Parreñas, 2020). Thus, along with broad trends towards familialism and informalization of care work, characteristic of liberal care regimes (Fagertun, 2017), some scholars suggests that there is evidence of convergence towards the ‘migrant in the market’ (Lightman, 2019; Van Hooren, 2012) model of employment across international care economies.

Lightman (2021) uses data from 2010–2014, examining twelve care economies. She finds evidence of similarities in the worker characteristics and working conditions of care workers across liberal and corporatist care regimes, while suggesting that familialistic and social democratic care regimes continue to display distinctive patterns of care provisioning. Duffy and Armenia (2021) examine 47 countries and find substantial variation in the size and characteristics of the care economies, noting that policy structures, as well as wealth, play a fundamental role, with immigrants typically clustered in lower wage healthcare occupations. Notably, however, neither study quantifies any distinctive outcomes of immigrant women care workers.

The current study recognizes that care regimes may have ongoing relevance for a variety of phenomenon – e.g. cultural norms around appropriate caring, funding models, or the division of care responsibilities within couples – suggesting the need for specificity in any claims regarding convergence. Thus, the current study's focus is solely on the employment and wages of immigrant women care workers. Using 2016 LIS data, I examine the care economy across 17 countries and four care regimes with the aim of documenting and examining patterns of care employment for immigrant women. Specifically, I assess whether there is financial devaluation of low status care work across countries and care regimes, and if this is most detrimental to women immigrants. By providing the most detailed cross-national quantitative analysis of immigrant women in care work to-date, data are provided on whether and how different care regimes continue to display distinctive trends in their care economies or if, instead, there is compelling evidence of converging economies of care with respect to immigrant women workers. Contextualized within the broader context of growing neoliberal market-orientations internationally, alongside shrinking welfare states and the devolution of care responsibilities to immigrant women of colour, the findings centre the employment and wages of intersectionally disadvantaged immigrant women employed in low status care (Parreñas, 2020; Tungohan, 2019). The following section outlines the research design and methodology applied.

## Methodology

This study aims to provide as broad an analysis as possible of the potential congruencies (or divergences) experienced by immigrant women in low status care work internationally. As such, as many countries as was methodologically and/or logically possible were included in the analyses, using the 2016 micro-data files (Wave X) from the LIS. The LIS harmonizes cross-sectional data from household-based national surveys to ensure comparability and provides among the best cross-national data available for comparisons of employment and income (Luxembourg Income Study (LIS) Database, 2023).

The 17 countries analysed were organized into four care regimes based on the policy structure of their care economy as of 2016 – again with a focus on the principal institution providing care – be it state, market, family or a combination. Countries excluded from the analysis either: (a) did not have the necessary occupation or industry variables available in their 2016 datafile to capture care work or did not have a variable for immigration (e.g. Canada, Greece, and Italy), (b) had these variables but they were coded in such a way that cross-national comparison was not possible (e.g. Mali, Paraguay, Peru), (c) had a sample of immigrants that was too small to allow for the desired analysis (e.g. Slovakia) or (d) were the only country with required data available in their care regime (this was notably the case for Spain, which is typically classified as a Southern European care regime, and which I ultimately excluded due to the lack of within-regime comparative data available).

Ultimately, data from seven countries (Australia, Switzerland, Ireland, Israel, Luxembourg, the United Kingdom and the United States) were classified as ‘liberal’ care regimes, four (Austria, Belgium, Germany and France) were categorized as ‘corporatist’, two countries (Denmark and the Netherlands) were classified as ‘social democratic’, and four countries (Czech Republic, Estonia, Poland and Serbia) were categorized as ‘central and eastern European’ care regimes. As noted previously, given a lack of consensus among scholars with regard to how to best classify countries into care regimes, my classificatory schema followed that of Van Hooren (2012), King-Dejardin (2019) and Lightman (2021). I nonetheless applied caution in analysing regime-level results in recognition of this variability.

The selected sample includes employed individuals aged 18–70 years (of typical working age) who had positive earnings. As the focus is on individuals employed in lower-status caring occupations, (e.g. personal care workers, healthcare assistants, domestic housekeepers, babysitters, and teachers’ aides), those in higher status occupations were excluded from the analyses – specifically managers, professionals, skilled agricultural, forestry and fishery workers, trades persons, machine operators, and individuals working in the armed forces (see Lightman (2021) for a more detailed discussion). The resulting per-country samples range from a low of 1759 in Ireland to a high of 32,544 in the United States.

## Operationalizing low-status care work

Care work was operationalized based on prior research focused on low wage work in health and education (e.g. Lightman, 2021), and in line with formative work by Budig and Misra (2010), who rely on both occupation and industry variables to specify the sample of care workers. Using an ‘industry approach to care’ (Duffy & Armenia, 2021, p. 6), I included both direct (face to face) and indirect (supporting) types of care work jobs using the LIS standardized industry variable (ISIC groupings) and identifying individuals employed in *education, health, residential care, social work, and domestic activities in private households*. Concurrently, as prior research has found that the wages and job satisfaction in care are tied to level of occupational professionalization (Budig & Misra, 2010; Lightman & Kevins, 2019), I excluded the previously selected care jobs done by professionals and managers (e.g. doctors, registered nurses, or hospital executives), and focused only on lower status caring jobs where women and immigrants are typically overrepresented, using the International Standard Classification of Occupations (ISCO-08) variable.

### Other relevant variables

The models measure who engages in care work and capture any associated wage penalties (or benefits) that accrue to immigrant women within lower-status caring jobs. The main independent variables thus capture (a) work in care (in reference to comparable non-professionals working in non-caring occupations) (b) immigrant status (in reference to people born inside of the country) and (c) women (in reference to men). Notably, the LIS does not provide a consistent measure of respondents’ race/ethnicity across national datasets, which would have been particularly illuminating for any intersectional analysis.

Controls were included wherever possible to capture family structure (specifically: age, marital status/cohabitation, and living with one's child aged 0–5 years) as well as educational attainment (measured using a categorical variable that is harmonized across the LIS datasets). In the descriptive analyses, additional variables capturing the precarious and nonstandard components of the care economy were also included where available (e.g. the proportion of care workers that are part-time, non-permanent, self-employed, and/or working in the private sector).

## Descriptive findings

Table 1 provides an initial impressionistic overview of the paid care economy, across countries and at mean levels by care regime. The aim here is to provide insight into the first research question regarding whether care economies are overall similar or different both across and within four care regimes and if countries increasingly provide a more ‘liberal-style’ of care provisioning with respect to heavy reliance on low skill immigrant workers. The first column examines the size of the care economy as a proportion of the total workforce. At average levels, it is immediately apparent that the proportions are similar in liberal, corporatist and social democratic regimes. Here, care work represents approximately 9–11% of the total labour force on average, with minimal variation across countries. By contrast, the CEE care regimes have far smaller care economies, on average, at roughly half the size, and comprising approximately 5% of the total labour force.^2^ The country where care comprises the largest component of the total economy is Germany (at 12.4%) and the smallest is Poland (at 3.4%).

**Table 1.** Overview of the paid care economy (low status work in health, education, social work, and domestic services), by country and care regime, 2016.

	N	% of Total Workforce	% of Low Status Workforce	% Women	% Immigrant	% Part- Time	% Non-Permanent/ Temporary	% Self-Employed	% Private Sector
**Care Regime**									
**Liberal (Mean)**	**8225**	**9.3**	**22.7**	**83.0**	**26.2**	**35.8**	**11.0**	**2.7**	**75.4**
Australia AU	5930	8.5	23.4	86.8	31.9	40.9	N/A	4.6	N/A
Switzerland CH	3472	10.8	24.4	80.4	32.7	37.6	11.9	0.9	N/A
Ireland IE	1759	8.16	20.2	81.7	17.9	37	11.2	4.8	N/A
Israel IL	4422	9.7	24.2	83.5	29.7	N/A	N/A	4.4	N/A
Luxembourg LU	2016	6.8	15	85.5	39.8	41.1	10.4	0.5	94.4
United Kingdom (UK)	7434	12.2	29.6	80.3	15	35.7	10.6	0.4	52.9
United States (US)	32,544	9.2	22	83.1	16.6	22.7	N/A	3.5	79
**Corporatist (Mean)**	**7654**	**10.9**	**24.6**	**80.5**	**15.4**	**43.7**	**14.4**	**6.0**	**64.6**
Austria (AT)	2399	9.4	20	80.1	20.8	43.2	7.8	N/A	80.9
Belgium (BE)	2240	9.3	22.5	79.5	12.5	50.5	11.2	N/A	62.5
Germany (DE)	8255	12.4	25.4	80.2	18.9	49.5	19.2	5.1	55
France (FR)	17,722	12.3	30.3	82.1	9.2	31.7	19.5	6.8	60.1
**Social Democratic (Mean)**	**14,622**	**11.4**	**28.4**	**82.6**	**10.7**	**79.0**	**13.2**	**3.3**	**N/A**
Denmark (DK)	26,379	11.3	29.4	83.1	11.5	N/A	N/A	1	N/A
Netherlands (NL)	2864	11.4	27.4	82	9.9	79	13.2	5.5	N/A
**Central and Easter European (Mean)**	**4946**	**5**	**13**	**85**	**7**	**8**	**12**	**3**	**25**
Czech Republic (CZ)	3279	4.9	12.1	87	0.8	9	13	5.3	N/A
Estonia (EE)	2065	5.2	16.5	93.1	18.1	11.8	1	0.5	28.1
Poland (PL)	12,428	3.4	10.9	85.8	0.3	8.6	22.7	3.6	30.3
Serbia (RS)	2013	4.9	14.3	73.7	7.1	3.8	11.2	2	16.6

*Note:* Population is limited to individuals aged 18–70, who are employed and have earnings >$0.

A similar trend of congruency is seen when examining care work as a proportion of the low status workforce only (recall, from above, this is defined here as individuals working as technicians or associate professionals, clerical support workers, service and sales workers, or in elementary occupations). This low status workforce is the focal population for all ensuing analyses, as these are considered ‘comparable workers’ to those engaged in low status care (versus those workers with higher occupational status, e.g. professionals and managers). Across the liberal, corporatist, and social democratic care regimes, care workers comprise greater than 20% of the low status workforce on average, with some variation across countries. The two social democratic countries demonstrate substantially higher proportions of care workers, at above 27%, while lower rates of 15% and 20.2% are seen in Luxembourg and Ireland, respectively. Again, the CEE countries have care as a far lower proportion of the low status workforce at approximately 13% on average.

Next, examining the focal demographic characteristics for this analysis, in terms of *gender*, there is striking convergence across all countries and care regimes. Upwards of 80% of low status care workers are women in sixteen of 17 countries (with Serbia as a slight outlier at 73.7% women in care). This reinforces the wealth of previous research documenting the highly feminized nature of low status work in care (e.g. Duffy & Armenia, 2021; Folbre, 2012; Turnpenny & Hussein, 2022). However, in examining *immigrant* makeup, there are notable differences. The proportion of immigrant workers in the care economy is by far the highest in the liberal regime countries, on average, at 26.2%. This is followed by the corporatist countries at 15.4%, on average. The comparatively high mean proportions in these two care regimes is unsurprising, given that liberal and corporatist countries have traditionally been primary immigrant-receiving nations, with robust policy frameworks to facilitate the entryway of foreign-born individuals, in some cases with specific pathways for those providing care (Banerjee, 2013; Tungohan, 2019). The social democratic countries, in comparison, have fewer immigrants working in their care economy (at 10.7% on average), reinforcing prior findings that due to their more robust welfare states, fewer immigrants work in care due to these jobs being better paid and more attractive to native-born workers (Lightman, 2021; Van Hooren, 2012). The care economy comprises still lower rates of immigrants in the CEE regime countries (besides Estonia, which stands out as an outlier at 18.1% immigrants). This may be tied to these countries generally having less immigration overall than in the comparison countries (World Bank, 2023).

Finally, the remaining columns in Table 1 provide the available data on job characteristics within the care regimes. Unfortunately, many countries have missing data here. However, what data exists shows trends of both variation and convergence across care regimes. For example, care workers are less likely to be part time in the CEE countries, while upwards of a third of care workers in liberal and corporatist regimes, on average, do not work full time. However, across countries and care regimes, care workers are unlikely to be self-employed or non-permanent/temporary workers. However, a major divide is seen across regimes in terms of the proportion of care workers located in the in private sector. Reinforcing prior research (e.g. Lightman, 2021; Van Hooren, 2012), the data show that care workers are far more likely to be working in the private sector in the liberal and corporatist regime countries where data is available, especially in Luxembourg, Austria, and the United States, while this is far less common in the CEE countries, a finding likely tied to the history of state-run social services in many of these nations (Bartha & Zentai, 2020; Schulz-Nieswandt et al., 2006).

**Figure 1.**
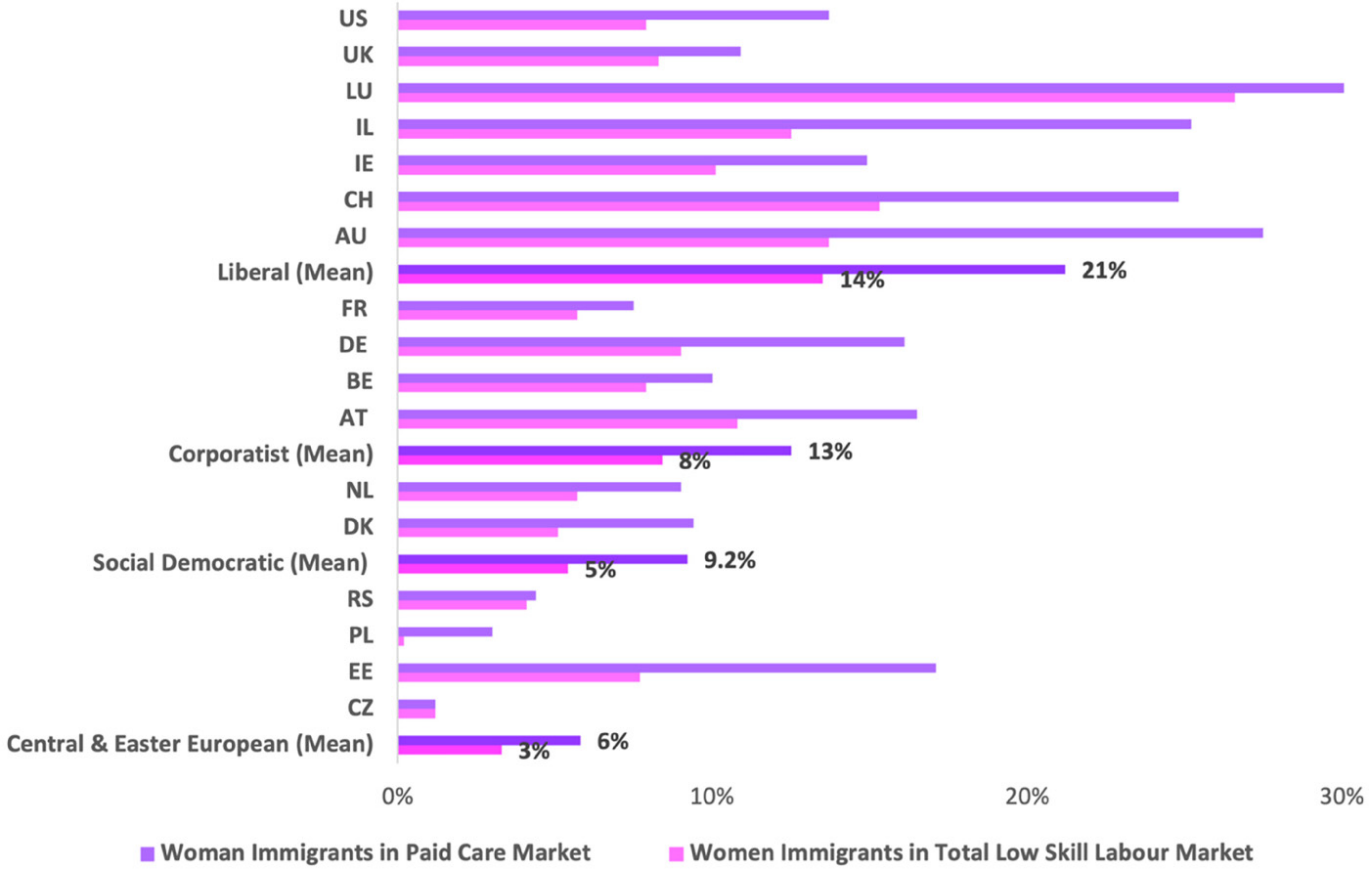
Comparison of women immigrants as a proportion of the paid care market and the low status workforce, by country and care regime, 2016.

Next, further descriptive detail on the ‘migrants in the market’ across countries and care regimes is provided to partially address the second research question, focused on the overrepresentation of immigrant women in low status care. Figure 1 compares the proportions of women immigrants working in care with those working in the entire low status workforce. Here there is conclusive evidence of the overrepresentation of these workers in low wage jobs in health, education, social work, and domestic services. In all 17 countries examined, women immigrants are overrepresented in care work. The largest differences are visible in liberal regime countries such as Australia (a 13.8% difference), Ireland (a 12.7% difference) and Switzerland (a 9.5% difference), with certain countries in the corporatist regime also demonstrating major overrepresentation, for example, Germany (a 7.1% difference) and Austria (a 5.7% difference).

The large share of immigrant women workers in the care market echoes what was seen in Table 1, which examined these ascriptive characteristics individually, rather than in combination. Thus, Figure 1 supports the suggestion that liberal and corporatist care regimes rely most heavily on women immigrants in the market to provide care, but the overrepresentation of these workers is also visible in the social democratic and CEE regimes.

**Figure 2.**
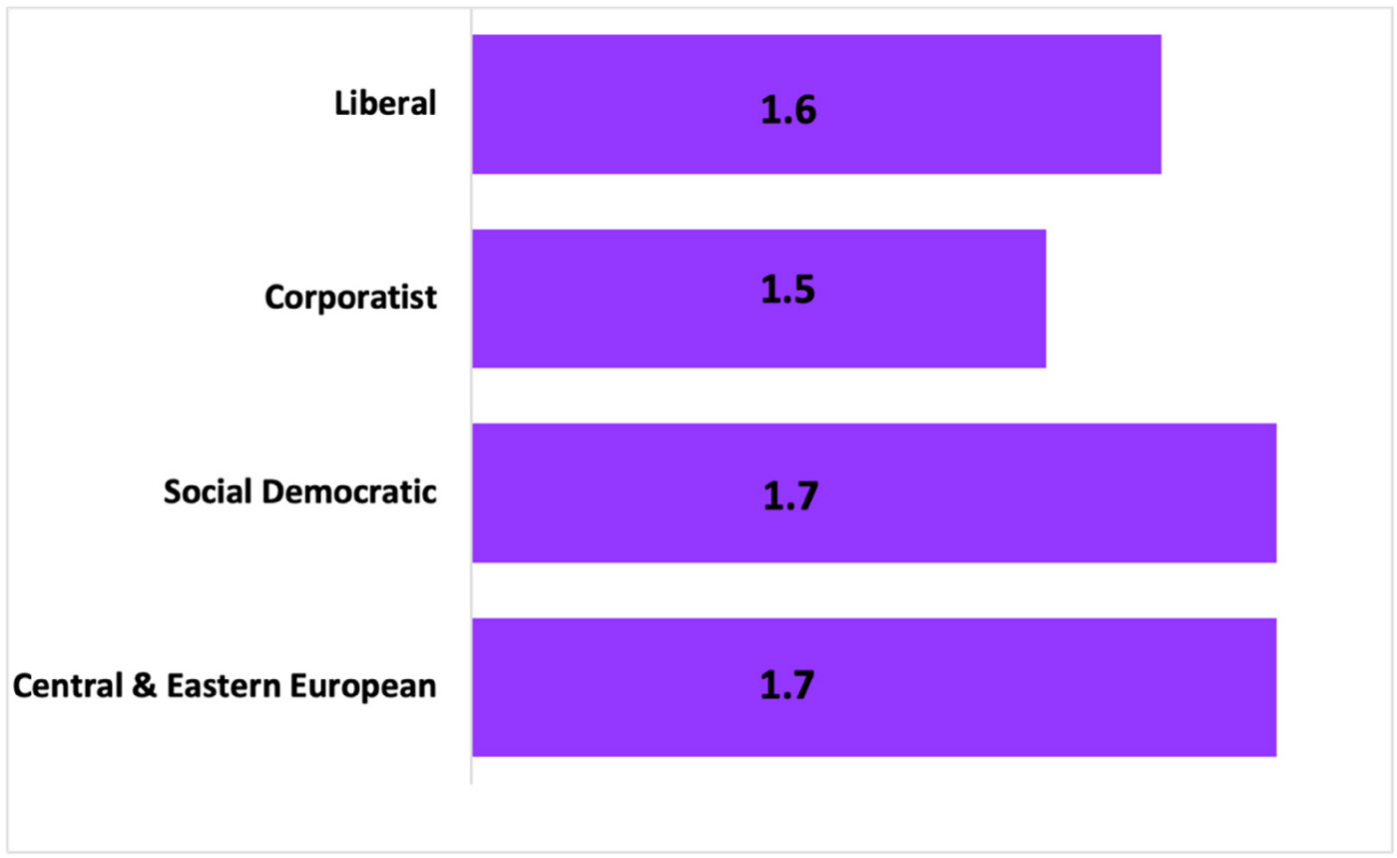
Ratio of women immigrants in the paid care market to women immigrants in the total low status workforce, by care regime, 2016.

As the final descriptive data, Figure 2 presents the mean ratio of women immigrants in the paid care market in comparison to in the total low status labour market by care regime (based on the data in Figure 1). Here, very clear and striking similarities across care regimes are revealed. The ratio is at 1.7 in the social democratic and CEE countries, at 1.6 in the liberal regime, and at 1.5 in the corporatist regime. Thus, while Table 1 and Figure 1 demonstrate that there remain substantial differences across care regimes, Figure 2 provides evidence supporting a care convergence with respect to immigrant women workers, as demonstrated by a consistent and substantive overrepresentation of women immigrants in the low status care workforce across care regime types.

Together, the descriptive data provide a range of results. On the one hand, Table 1 suggests that intragroup variation is minimal within the care regimes examined, but cross-group variation remains substantial. This is supported by the wide variation in the overrepresentation of immigrant women workers across care regimes as seen in Figure 1. Yet, in combination, Figure 1 and Figure 2 suggest trends towards a ‘migrant in the market’ model of employment, with women and immigrants highly overrepresented in low wage care jobs in all the 17 countries examined. Thus, while variation within care regimes is evident on some measures – for example, the size of the care workforce, and the proportion of care workers that are part-time workers or located in the private sector – general trends towards convergence with respect to immigrant women workers are apparent in 2016. Notably, women immigrants are demonstrated to be the backbone of the care industry, regardless of regime type. However, these data beg the question of whether the overrepresentation of immigrant women in low status care work is primarily a function of their lower educational attainment, something that is examined in the ensuing regression analyses.

## Multivariate findings

The multivariate analyses present models that control for educational attainment and demographic characteristics (age, marital status/cohabitation, and the presence of young children in the household) to isolate any effects of the independent variables. To address the second component of research question #2, examining if there is overrepresentation of immigrant women in care work net of educational attainment, Table 2 presents the chances out of 100 of performing low status paid care work (for low status workers) for the four combinations of workers associated with gender and immigration status (women immigrants, men immigrants, women non-immigrants, and men non-immigrants). This is based on results from logistic regression models with interactive coefficients, run across countries and at mean levels for each care regime.

**Table 2.** Chances out of 100 of performing low status paid care work (for low status workers), by country and care regime, 2016.^(a)(b)(c)^

	Liberal								Corporatist				
	AU	CH	IE	IL	LU	UK	US	Mean	AT	BE	DE	FR	Mean
**Female immigrant**	35.4	31	23	37.2	18.7	33.6	32.1	**30.1**	30	29.2	39.6	29.7	**32.1**
**Female non-immigrant**	30.9	33.8	25.9	36.6	27.2	36.9	30	**31.6**	25.2	31.4	32.2	36.7	**31.4**
**Male immigrant**	10.7	14	6.4	12.5	6.6	21.3	9.1	**11.5**	10.2	9.5	12.9	14.1	**11.7**
**Male non-immigrant**	10.9	10.3	9.8	9.9	3.3	15.6	9.3	**9.9**	9.2	11.1	12.9	15.5	**12.2**
***n***	4693	3472	1730	3367	2009	7225	32,544	**7863**	2399	2141	8194	17,073	**7452**

**Table:** 

	Social Democratic			Central and Eastern European		
	DK	NL	Mean	EE	RS	Mean
**Female immigrant**	49.8	37.8	**43.8**	22.2	11.1	**16.7**
**Female non-immigrant**	38.2	37.9	**38.1**	21.2	18.4	**19.8**
**Male immigrant**	18.9	7.3	**13.1**	2.7	9.3	**6.0**
**Male non-immigrant**	11.9	11.8	**11.9**	3.3	7.7	**5.5**
***n***	24,296	2848	**13,572**	2065	2013	**2039**

*Note:* Population is limited to individuals aged 18–70, who are employed and have earnings >$0.

(a) Logistic regression results control for educational attainment and demographic characteristics (age, marital status/cohabitation, and the presence of young children in the household).

(b) Divided by 100 the product is a probability

(c) CZ and PL not estimable because of collinearity issues tied to the very small sample size of male immigrants in low wage care work occupations

Overall, the data in Table 2 demonstrate that once educational attainment and the other relevant demographic factors are controlled for, in the majority of countries analysed the differences in terms of the probabilities of working in care work between immigrant women and non-immigrant women diminish or are eliminated. Analysed in tandem with Figures 1 and 2, this suggests that it is, in fact, due to variations in educational attainment and demographic variables that we see major disparities in terms of the overrepresentation of immigrant women in care, as compared to non-immigrant women. This builds on prior research suggesting that vulnerable individuals are often negatively selected into care occupations based on low levels of education, resulting in lower wages (England et al., 2002; Lightman, 2019).

Table 2 provides evidence of some variation across the liberal countries, with women immigrants having the highest chance of working in care in Australia (at 35.4 chances out of 100) and the lowest chance in Ireland (at 23 chances out of 100). Non-immigrant women have the highest chances of working in care in the United Kingdom (at 36.9 chances out of 100) and the lowest chance again in Ireland (at 25.9 chances out of 100). In examining the corporatist countries, the trend is strikingly similar, with the highest chance for immigrant women of working in care in Germany (at 39.6 chances out of 100) and the lowest in France (at 29.7 chances out of 100). When examined at mean levels, the probabilities are also very similar in the liberal and corporatist care regimes for non-immigrant women (at 30.1 and 32.1 chances of out 100, respectively). Thus, at mean levels there are very similar probabilities of working in care for comparable immigrant and non-immigrant women in the liberal and corporatist care regimes, again suggesting a trend of convergence.

Examining the other countries, we see that in the social democratic regime countries women have the highest probability of working in care when controlling for education – both for immigrant women (at 43.8 chances out of 100 on average) and non-immigrant women (at 38.1 chances out of 100 on average). This finding is likely again related to the highly developed welfare state characteristic of social democratic countries, leading to more jobs in the care economy that are attractive to non-immigrant workers. In the CEE regime countries, where data are available there are lower probabilities of working in care for both groups of women with a slightly higher chance out of 100 for non-immigrant women (at 19.8) than immigrant women (at 16.7) on average.

Finally, examining the chances out of 100 of working in care for men, Table 2 demonstrates that in all countries and regime types, men are far less likely to work in care than comparable women (typically one half to one third as likely), regardless of immigration status. Yet a similar trend is seen for women and men, as in only 7 of 16 countries do immigrant men and women have a (in many cases very slight) higher probability of working in care than non-immigrant men or women, when controlling for educational attainment. Thus, Table 2 not only demonstrates the highly feminized nature of low wage care work across countries and care regimes, but also suggests that while immigrant women are overrepresented in this work in all countries examined, this difference is likely tied to educational attainment and demographic factors in slightly more than half of the cases examined. There is again evidence of convergence across the liberal and corporatist care regimes, but within regime variation is also apparent.

**Figure 3.**
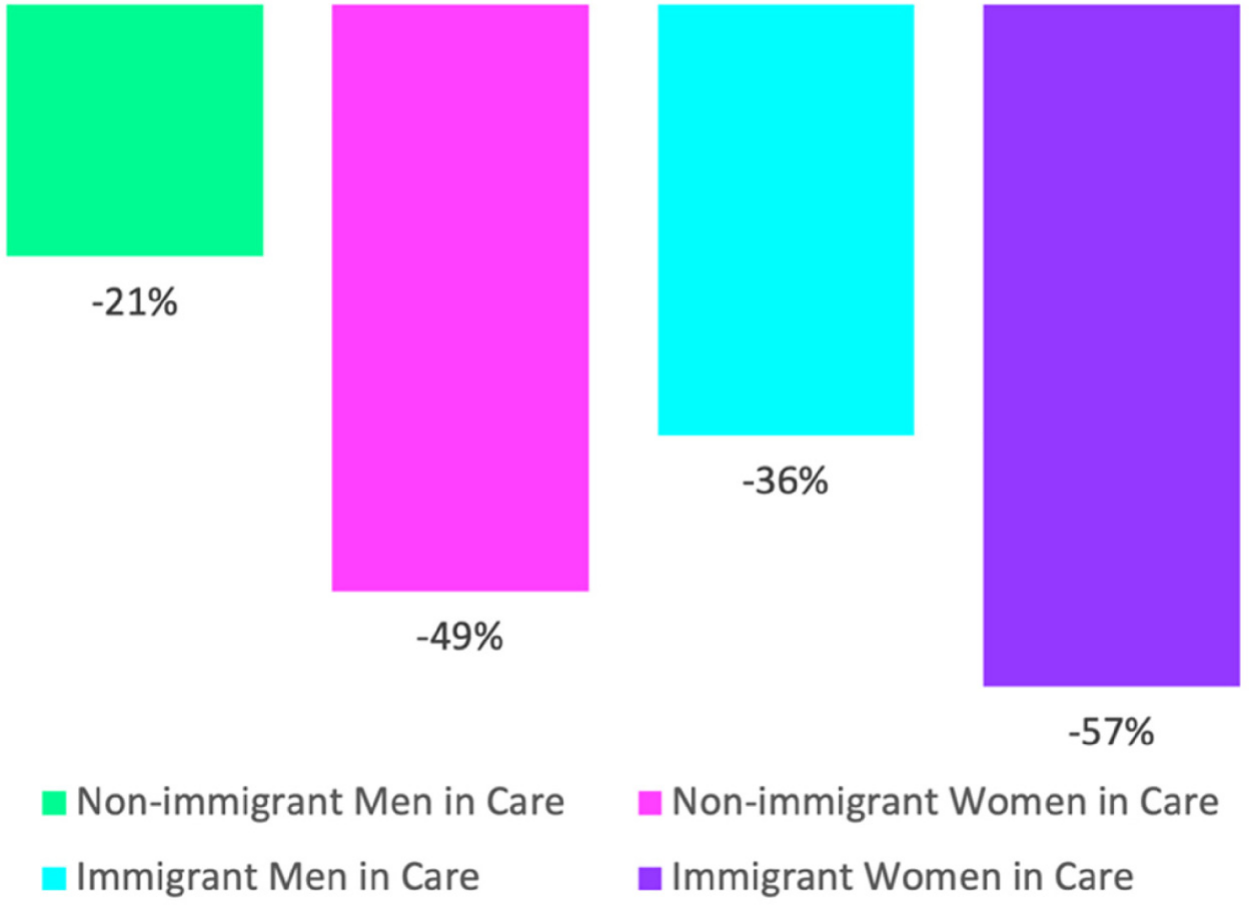
Significant wage penalties for groups working in paid care (relative to non-immigrant men working outside of care in the low status workforce), 2016.

Finally, Table 3 and Figure 3 provide data to address the third research question, measuring any wage penalties (in percentage points) experienced by care workers, women, and immigrants, relative to other low status workers, by country and care regime. This provides a means to examine potential disparities tied to these factors. In Table 3, results are provided from individual country and regime-level OLS regressions, with the dependant variable logged individual income and controlling for all educational attainment and demographic variables. Significant results are bolded. From Table 3, it is notable that in all countries where the effect is significant there is a wage penalty for work in care ranging from a low of −5.9% in Switzerland to a high of a full −31.9% in Ireland. The mean wage penalty for work in care is similar across the liberal and corporatist care regimes at −12–13%. It is slightly lower in the social democratic regime (at approximately −7%), on average, and it is smallest in the CEE regime (at −2.9%), on average. This latter finding is partially due to non-significant wage bonuses in some CEE regimes countries, as well as overall lower wages across the low status economy in central and eastern Europe. However, Table 3 provides compelling evidence that bolsters prior research documenting significant and substantive wage penalties in low status care work (e.g. Budig & Misra, 2010; Folbre, Gautham & Smith, 2021).

**Table 3.** Percentage point differences in logged annual earnings for care workers, women, and immigrants (relative to other low status workers), by country and care regime.^(a)(b)^

Wage Penalties			
	Care Work	Women	Immigrants
**Liberal (Mean)**	**−13.1**	**−38.5**	**−9.4**
AU	−3.4	**−40.9**	**−7.9**
CH	**−5.9**	**−42.5**	**−7.3**
IE	−1.0	**−37.6**	**−20.3**
IL	**−31.9**	**−37.0**	−4.3
LU	**−19.3**	**−37.2**	**−7.2**
UK	**−20.0**	**−37.2**	**−8.3**
US	**−10.5**	**−37.3**	**−10.5**
**Corporatist (Mean)**	**−12.4**	**−35.3**	**−18.7**
AT	−6.9	**−48.2**	**−33.6**
BE	**−11.2**	**−23.2**	−6.4
DE	**−16.9**	**−40.6**	**−21.1**
FR	**−14.7**	**−29.2**	**−13.8**
**Social democratic (Mean)**	**−7.2**	**−21.7**	**0.0**
DK	**−10.9**	+0.5	**+1.0**
NL	−3.5	**−43.9**	**−**1.0
**Central and Eastern European**	**−2.9**	**−32.1**	**−2.6**
CZ	**−11.1**	**−38.1**	−9.5
EE	6.7	**−48.5**	**−12.2**
PL	**−8.0**	**−28.4**	11.2
RS	0.7	**−13.4**	0.2

Notes: The sample is limited to employed individuals aged 18–70 and who have earnings greater than $0.

(a) Significant effects (*p* < .05) are bolded.

(b) Results control for the number of children, age, age squared, marital status, and level of education.

Next, Table 3 demonstrates the ongoing negative effect of being a woman in the low status labour market, again across countries and care regimes. Being a woman is associated with the largest wage penalty in all countries where gender is significant (controlling for work in care and immigration status), ranging from a ‘low’ of −13.4% in Serbia to a high of a full −48.5% in Estonia. The mean values are again strikingly similar in the liberal and corporatist care regimes and well as in the CEE regime (at −38.5%, −35.3% and −32.1%, respectively), with lower penalties in the social democratic regime, on average, again providing evidence of the effectiveness of the defamilialization of the labour market in bolstering women's wages (Giordano, 2022; Van Hooren, 2012). Finally, there is substantial variation in the wage penalties experience by immigrants, with only Denmark demonstrating a slight 1% wage bonus for immigrant workers (controlling for gender and work in care). The wage penalty for immigrants is on average −9.4% for the liberal regime countries, slightly lower than the associated wage penalty for work in care, while the reverse is true in the corporatist regimes where the penalty for immigrants (at −18.7%) is slightly greater than that for care work. For the CEE countries the mean effect for immigrants is similar (and overall smaller as compared to the other regimes) to that for care work (at −2.6%), again suggesting smaller wage variations overall.

Thus, Table 3 not only suggests substantial variation across countries and in some cases across care regimes, but also demonstrates the major cumulative wage disadvantage experienced by workers with intersectional disadvantages, as care workers, women, and immigrants (aka ‘migrants in the market’). Wage penalties are overall similar in liberal and corporatist care regimes, with differences across and within the other regimes, suggesting that care regimes may still have heuristic utility as a categorization schema, when it comes to relative wage devaluation of work in care, notwithstanding trends towards a liberal-style model.

As the final data provided, and to answer the second component of research question #3, a pooled country model is run to predict logged earnings among low status workers with interactions for gender and care work and gender and immigration status, while controlling for care regime and country fixed effects. For ease of interpretation, the results from the main effects and interactions are presented additively in Figure 3 (with the regression table provided in Appendix 1). Net of educational attainment and other demographic factors, as well as regime and country-level variation, Figure 3 demonstrates that the largest cumulative wage penalty is experienced by immigrant women in care (at a full −57% wage disadvantage compared to native-born men working outside of care in the low status workforce) followed by non-immigrant women in care (at −49%), immigrant men in care (at −36%) and non-immigrant men in care (at −21%). Thus, Figure 3 provides evidence of a clear hierarchy for groups working in low status care, with significant and very substantial wage disadvantages relative to the reference group. This figure provides substantiation to the suggestion that wage penalties in care work particularly disadvantage women immigrants, even when controlling for country-level variation and care regime type, and furthering evidence of a transnational convergence in the care economy with respect to the devaluation of immigrant women care workers.

## Conclusions: trending towards a care convergence with respect to immigrant women workers

This study began by examining two opposing perspectives on the ongoing utility of care regimes for examining care economies cross-nationally. The first suggests that care regimes are a meaningful heuristic device to classify and compare care markets across diverse countries and regions along patterns of similarity and difference. The second suggests that due to the predominance of the global care chain there has been an international convergence towards the employment of immigrant women in low status low wage care work, tied to neoliberal cuts to social service provisioning, and thus limiting the ongoing utility of care regimes as a classificatory measure. This latter trend has been effectively characterized by Van Hooren (2012) as a ‘migrant in the market’ model of employment.

Inevitably, a single empirical study such as this one cannot conclusively settle the debate as to whether care regimes remain relevant and useful for cross-national comparisons of care economies, or, instead, are an outdated typology given global convergence towards a ‘liberal’ style of care provisioning. However, the data provided herein, analysing 17 national care economies, is the largest scale analysis provided to date that focuses specifically on stratification within care markets, and on the disproportionate disparities experienced by women immigrant care workers in low status work in health, education, social work and domestic services across four care regimes.

The findings suggest some major similarities across liberal, corporatist, social democratic, and central and eastern European care regimes, in terms of the overrepresentation of immigrant women in low status care work, and the disproportionate financial penalties these workers incur. Addressing my first research question, the descriptive data demonstrate that care work is a substantial component of the low status economy for all countries examined, but also reveal ongoing variation across care regimes. Liberal and corporatist care regimes, comprising countries that have traditionally been major immigrant-receiving destinations, have more of a reliance on immigrant workers than social democratic or CEE regimes. Thus, there is mixed evidence of distinctiveness both within and across care regimes when gender and immigration status are looked at separately.

However, when the focus is on immigrant women in particular (addressing research question #2), similar trends vis-à-vis their substantial overrepresentation in care jobs across care regimes are evident (at a ratio of 1.5–1.7). Given the current context of continued privatization and austerity measures across the Global North and South, it thus seems likely that the global care chain will continue to expand, and facilitate ongoing flows of precarious, low-wage and temporary labour to provide care for wealthier segments of the population in social democratic and CEE countries, furthering existing trends in liberal and corporatist care regimes towards a ‘migrant in the market’ model.

The multivariate analyses examined the probability of working in low wage care for immigrant women, controlling for educational attainment (addressing the second component of research question #2). In these models, disparities between immigrant and non-immigrant women diminished or were eliminated in the majority of countries examined. This suggests that immigrant women are often negatively selected into care occupations based on low levels of education and human capital. It would seem, then, that the global care chain effectively filters immigrant women with low levels of education into jobs that are increasingly characterized by precarious conditions and have limited upward mobility. The implications of this trend for social integration and social solidarity over time are in critical need of further in-depth study.

Finally, the analyses of wage disparities in care work provide perhaps the most compelling evidence of both the independent and cumulative/interactive disadvantages consistently tied to gender, immigration status and work in care across countries and care regimes. Addressing the third and final research question, care workers are found to experience a wage penalty in all countries where results are significant. And, perhaps most crucially for the purposes of this study, immigrant women care workers are found to make a full fifty-seven percent less than comparable male, non-immigrant workers in low status occupations outside of the care economy, net of country and regime-level variation and educational attainment. The data thus demonstrate a clear hierarchy in terms of care wage penalties for workers at the intersection of gender and immigration status, notwithstanding specificities tied to individual care economies or regime types. This supports prior calls by care work scholars for more policy-specific comparisons of countries in terms of social support and care provisioning, while recognizing the predominance of the global care chain (e.g. Michel & Peng, 2017; Ranci et al., 2021).

Together, the findings contribute to two substantive areas in the empirical scholarship on care. First, regarding debate as to the ongoing utility of care regimes with respect to immigrant women workers, the data from 17 countries suggest that there are large-scale trends towards convergence across economies of care. While the data do support prior research suggesting that the greatest evidence of a ‘migrant in the market’ model is seen across liberal and corporatist regime countries (Lightman, 2021; Van Hooren, 2012), the social democratic and CEE countries also demonstrate substantial similarities in terms of the wage devaluation of care workers and the overrepresentation of immigrant women workers. Second, this study highlights the importance of looking at interactions of immigration and gender (as well as race) in quantitative analyses of care workers, rather than examining isolated effects of specific traits. Looking at immigration or gender (or race) in isolation within the care economy leaves intersectional disadvantages muted or ignored, and thus minimizes the data's utility for effective policy interventions.

Finally, the LIS data analysed has notable limitation. As a result, the findings support calls for more detailed analyses of care work and care workers in cross-national comparison. Such studies would benefit from: (a) data that can account for variations in immigration statuses, to examine workers who are permanently resettling in the countries they provide paid care versus those who are on temporary visas, and to encompass those who work legally as well as those who are irregular (b) data that includes individuals working in the informal or grey care market, which is not captured in the LIS dataset but encompasses a substantial component of the care economy in the countries examined. Such work typically employs racialized and immigrant women in disproportionate numbers; (c) data that can measure specific differences tied to racialization, recognizing that stratification tied to race is essential for a fulsome analysis of low status care work; (d) qualitative studies that can delve deeply into intersectional disparities for specific subgroups within the care economy, and finally, (e) given the limitations of looking at the entire care market altogether, which necessarily leads to a broader but more superficial analysis, there is a need for data that facilitates more detailed comparisons of the implications of specific care policies, for example, studies that focus solely on the provision of institutional long-term care, homecare, or childcare in private homes. All of these additions to the existing cannon of care work scholarship would provide fruitful insights regarding differences and similarities across and within care regimes, as well as further illuminating who can afford care, who can access it, and where and how it is provided internationally.

It is hoped that the present analysis, along with future cross-national studies of care work, will contribute to ongoing debate as to the benefit of care regimes for comparative analyses of immigrant women workers, as well as providing policy-relevant data to inform decision-making. Individual countries and/or jurisdictions require comparative analyses to improve working conditions for care workers, as well as the quality of care provided to populations in need. Without a dedicated effort in the policy realm to improve the care economy for both workers and clients, it seems likely that the trend towards neoliberal and privatized care provisioning will continue, at the expense of immigrant women workers, and with reduced accessibility of social care for intersectionally disadvantaged individuals in need.

## Notes

1. Nancy Folbre defines the ‘care economy’ as encompassing ‘expenditures on children and other dependents; paid care work; and unpaid care of family, friends, and neighbors’ (Folbre, 2018, p. 6). The focus of this article is solely on low wage paid care work within the care economy.

2. Notably, these are also countries that have comparatively smaller GDPs as of 2016 (World Bank, 2023).

## Appendix Table 1: Pooled Country Model Predicting Logged Earnings among low-status workers (Controlling for Care Regime and Country Fixed Effects), 2016.

**Table:**

	Coefficient	S.E.
Intercept	**8.31**	(0.04)
Care effect (ref = non-care employment)	**−0.21**	(0.02)
Immigrant status (ref = non-immigrant)	**−0.15**	(0.01)
Female (ref = male)	**−0.38**	(0.00)
Immigrant × care work	**0.07**	(0.02)
Female × care work	**0.10**	(0.01)
Age	**0.09**	(0.01)
Age squared	**−0.01**	(0.00)
Married or co-habitating (ref = single/widowed/divorced)	**0.10**	(0.01)
Education level (ref = low)		
Medium	**0.28**	(0.01)
High	**0.58**	(0.01)
Statistical fit		
Adjusted R square	0.42	
*n* (Countries)	17	
*N* (Individuals)	136,538	

*Note:* Population is limited to individuals aged 18–70, who are employed, and have earnings >$0.

Significant effects (*p* < .05) are bolded.
